# Views and Perceptions of Amyloid Imaging in a Preclinical Alzheimer’s Disease Trial

**DOI:** 10.14283/jpad.2024.157

**Published:** 2024-09-03

**Authors:** Marina Ritchie, R. Raman, K. Ernstrom, S. Wang, M. C. Donohue, P. Aisen, D. Henley, G. Romano, G. P. Novak, H. R. Brashear, R. A. Sperling, J. D. Grill

**Affiliations:** 1 UC Irvine Institute for Memory Impairments and Neurological Disorders (UCI MIND), 3230 Biological Sciences III, University of California, Irvine, Irvine, CA USA; 2 Alzheimer’s Therapeutic Research Institute, University of Southern California, San Diego, CA USA; 3 Janssen Research & Development, LLC, Titusville, NJ USA; 4 Indiana University School of Medicine, Indianapolis, IN USA; 5 University of Virginia, Charlottesville, VA USA; 6 Brigham and Women’s Hospital, Harvard Medical School, Boston, MA USA

**Keywords:** Biomarker disclosure, recruitment, preclinical Alzheimer’s disease, clinical trials

## Abstract

**Background:**

Many cognitively unimpaired older adults are interested in learning their Alzheimer’s disease (AD) biomarker status, but little is known about motivations to undergo biomarker testing and result disclosure in the setting of preclinical AD trials.

**Objectives:**

Examine whether motivations to undergo AD biomarker testing and disclosure differ for individuals who have elevated amyloid compared to those with not elevated amyloid, and whether disclosure of amyloid results impacts participants’ motivations.

**Design, Setting, Participants:**

We conducted post-hoc analyses using data from the EARLY study, a preclinical AD trial of the beta-secretase inhibitor atabecestat. As part of the screening process of the trial, participants underwent biomarker testing and disclosure. We analyzed data from n=2241 participants.

**Measurements:**

We analyzed data from the Views and Perceptions of Amyloid Imaging (VPAI), a 9-item questionnaire assessing how strongly participants agreed with motivating factors for undergoing amyloid testing. The VPAI was administered at the first screening visit and again after amyloid disclosure.

**Results:**

Prior to amyloid disclosure, a greater proportion of participants in the elevated amyloid group responded at the two highest levels of endorsement for the items, “to confirm the feeling that I might already be developing symptoms of AD dementia” (p<0.001) and “to prepare my family for my possible illness in the future” (p=0.008), compared to participants in the not elevated amyloid group. Following disclosure, the not elevated amyloid group had higher odds of positive change in categorical VPAI item level scores for the items “to put mind at ease” (OR: 0.54; p<0.001), “to confirm the feeling that I might already be developing symptoms of AD dementia” (OR: 0.79; p=0.049), and “to prepare my family for my possible illness in the future” (OR: 0.67; p=<0.001), while the elevated amyloid group had higher odds of positive change for the item “curiosity” (OR:1.32; p=0.014).

**Conclusions:**

Investigators might consider adjusting recruitment strategies for future trials to align with the motivations to undergo biomarker testing and disclosure most strongly endorsed by participants with elevated amyloid.

**Electronic Supplementary Material:**

Supplementary material is available in the online version of this article at 10.14283/jpad.2024.157.

## Introduction

**I**nadequate trial recruitment is a consistent barrier to Alzheimer’s disease (AD) drug development, and there are unique challenges at the preclinical stage of disease (1, 2). First generation preclinical AD trials required participants to undergo amyloid positron emission tomography (PET) scans or lumbar punctures and learn their biomarker result as a component of learning their trial eligibility. Though many cognitively unimpaired older individuals have expressed interest in learning their biomarker results in large surveys (3–5), relatively little is known about motivations to undergo amyloid biomarker testing and disclosure among participants who enroll in preclinical AD trials. Further, whether these motivations differ for individuals who meet criteria for preclinical AD, compared to those who do not, and whether biomarker disclosure affects participant’s motivations, remain relatively understudied. Given the high screen failure rates based on amyloid biomarker status, identifying potential differences in reported motivations between groups could help trialists prioritize the motivations endorsed by those more likely to be eligible.

Much of the available data on attitudes toward preclinical AD trials and undergoing amyloid imaging has come from studies using hypothetical questions, focus groups, and from one preclinical AD trial: the Anti-Amyloid Treatment in Asymptomatic Alzheimer’s disease (A4) study (6–8). In this study, we examined these constructs in another preclinical AD trial—the EARLY trial (ClinicalTrials.gov identifier: NCT02569398). The EARLY trial tested the safety and efficacy of the nonselective BACE inhibitor atabecestat (9, 10) in preclinical AD. We analyzed the data collected with the Views and Perceptions of Amyloid Imaging (VPAI) scale during the screening process to examine why older cognitively unimpaired individuals chose to enroll and undergo AD biomarker testing in this study.

## Methods

### Data source

The EARLY trial was a phase 2b/3 trial of the nonselective oral β-secretase inhibitor, atabecestat, conducted from November 2015 to December 2018 (10, 11). The trial was terminated prematurely due to treatment related safety concerns (9). Participants were enrolled in 10 countries. All participants signed the IRB-approved trial consent form, which included consent to secondary analyses such as those included in this study. The current analysis did not meet the criteria for human subjects research, as no identifiable information was used.

Cognitively unimpaired participants with a Clinical Dementia Rating (CDR) global score of 0, with or without subjective cognitive concerns were enrolled in the trial (10). Participants were required to undergo biomarker testing for elevated brain amyloid via PET or cerebrospinal fluid (CSF) as well as disclosure of their biomarker result as part of the screening process for the EARLY study. The disclosure process was similar to a previous preclinical AD trial (12). There were 5 screening visits all of which were completed within 90 days before randomization (10). Education and counseling were performed at consent; biomarker testing was performed on a separate day from consent, and disclosure was performed on a separate day from biomarker testing. Participants were informed that they had either an elevated or a not elevated amyloid biomarker result (9).

### Primary outcome measure

VPAI is a participant-reported questionnaire with 9 items that assess reasons for undergoing amyloid imaging. VPAI was adapted from an existing questionnaire (13) for use in preclinical AD trials. Each item is scored on a 5-point Likert scale, with 1 indicating “not at all” and a 5 indicating “extremely” important. Participants were asked to complete the VPAI at the first screening visit (pre-disclosure) and on the day of the disclosure after learning their amyloid result.

### Statistical analyses

To understand why older individuals undergo biomarker testing in the setting of a preclinical AD trial, we first examined VPAI responses collected before biomarker testing and disclosure. We examined the frequency and percentage with which participants endorsed reasons for undergoing amyloid biomarker testing and disclosure across all participants; we then examined whether this differed between amyloid groups. We used chi-squared tests for comparisons between the elevated and not elevated amyloid groups in the frequency with which participants responded at the two highest levels (“very” or “extremely” important) for each of the VPAI items. Holm’s adjusted p-values were computed to account for the number of comparisons. We a priori chose to dichotomize the VPAI responses due to the small sample sizes in the extreme categories and to operationalize meaningful endorsement scores and changes in scores.

We explored potential changes in VPAI responses after biomarker testing and disclosure. To do so, we categorized VPAI item level pre- and post-disclosure scores as 0 if the VPAI item level score was 1–3 (“not at all,” “a little,” “somewhat” important) and 1 if the VPAI item level score was 4 or 5 (“very” or “extremely” important). Changes in this binary VPAI score (VPAI item level post - pre scores) were computed and labeled as Reduced (−1), No Change (0) or Increased (1). For each VPAI term, we used a generalized estimating equations (GEE) model with a logistic link function to assess the relationship between amyloid group and the binary pre-and post-disclosure VPAI item response (‘extremely’ or ‘very’ important versus ‘somewhat’ or ‘a little’ or ‘not at all’ important), adjusting for age, sex, and family history of AD (14). The advantage of the GEE model is that it accounts for the within-participant correlation. No adjustments were made for multiple comparisons due to the exploratory nature of these analyses.

As secondary analyses, we a priori assigned the 9 VPAI items into 4 thematic categories based on face validity and their use in a previous study, including perceived risk, altruism/contribute to research, plan/prepare, and curiosity (Table 1) (8). Item scores within each category were summed to create a total thematic category score. For each thematic category score, we fit a linear regression model for pre-disclosure and pre- to post-disclosure change scores, respectively. The dependent variable in the model was the category score and the predictor of interest was amyloid status. We adjusted the model for age, sex, and family history of AD. Linear regression assumptions were assessed using diagnostic plots (data available upon request).

**Table 1:** Views and Perceptions of Amyloid Imaging thematic categories

Category	Items
Perceived Risk	Item 2: To put my mind at ease if I found out I do not have elevated amyloid on my PET scan
	Item 7: To confirm the feeling that I might already be developing symptoms of Alzheimer’s disease dementia
Altruism and contribute	Item 4: To be able to participate in anti-amyloid clinical trials
	Item 5: The desire to contribute to research on Alzheimer’s disease
Plan and prepare	Item 1: To seek information on preventative measure (e.g., change diet, exercise, or other lifestyle changes)
	Item 6: To arrange my personal affairs
	Item 8: To prepare my family for my possible illness in the future
Curiosity	Item 3: To know more about my risk of developing Alzheimer’s disease dementia
	Item 9: Curiosity

All analyses were conducted using the statistical software R (version 4.3.0) (15).

## Results

### Participant characteristics

VPAI data were available for n=2241 participants who underwent biomarker testing and disclosure (Table 2). Demographic characteristics including age, sex, race and ethnicity, as well as the study partner’s relationship to the participant (study partner type), were comparable between the elevated and not elevated amyloid groups. There was a higher proportion of participants who were APOE4 carriers in the elevated compared to the not elevated amyloid group. No clear differences were observed between groups in family history of AD and dementia.

**Table 2:** Study participant demographics and clinical data

**Participant characteristics**	**Not Elevated (N= 1724)**	**Elevated (N= 517)**	**Total (N=2241)**
Age, mean (SD)	68.30 (5.19)	70.42 (5.56)	68.79 (5.35)
Sex Female, n (%)	1088 (63.1%)	337 (65.2%)	1425 (63.6%)
Race, n (%)			
American Indian or Alaskan Native	0 (0.0%)	0 (0.0%)	0 (0.0%)
Asian	15 (0.9%)	2 (0.4%)	17 (0.8%)
Black or African American	31 (1.8%)	5 (1.0%)	36 (1.6%)
Native Hawaiian or other Pacific Islander	0 (0.0%)	0 (0.0%)	0 (0.0%)
White	1658 (96.2%)	509 (98.5%)	2167 (96.7%)
Other	16 (0.9%)	1 (0.2%)	17 (0.8%)
Multiple	3 (0.2%)	0 (0.0%)	3 (0.1%)
Unknown	0 (0.0%)	0 (0.0%)	0 (0.0%)
Not reported	1 (0.1%)	0 (0.0%)	1 (0.0%)
Ethnicity, n (%)			
Hispanic or Latino	73 (4.2%)	19 (3.7%)	92 (4.1%)
Not Hispanic or Latino	1637 (95.0%)	495 (95.7%)	2132 (95.1%)
Not reported	10 (0.6%)	1 (0.2%)	11 (0.5%)
Unknown	4 (0.2%)	2 (0.4%)	6 (0.3%)
Country, n (%)			
AUS	243 (14.1%)	61 (11.8%)	304 (13.6%)
BEL	93 (5.4%)	23 (4.4%)	116 (5.2%)
CAN	6 (0.3%)	8 (1.5%)	14 (0.6%)
ESP	110 (6.4%)	25 (4.8%)	135 (6.0%)
FIN	20 (1.2%)	4 (0.8%)	24 (1.1%)
GBR	348 (20.2%)	64 (12.4%)	412 (18.4%)
ITA	20 (1.2%)	8 (1.5%)	28 (1.2%)
NLD	90 (5.2%)	25 (4.8%)	115 (5.1%)
SWE	19 (1.1%)	12 (2.3%)	31 (1.4%)
USA	775 (45.0%)	287 (55.5%)	1062 (47.4%)
APOE, n (%)			
Carrier	426 (25.8%)	282 (55.7%)	708 (32.8%)
Non-carrier	1125 (74.2%)	224 (44.3%)	1449 (67.2%)
Missing	73	11	84
Family History of AD (Yes), n (%)	730 (42.3%)	250 (48.4%)	980 (43.7%)
Study partner type, n (%)			
Spouse	1146 (67.4%)	339 (65.7%)	1485 (67.0%)
Adult child	165 (9.7%)	57 (11.0%)	222 (10.0%)
Other	390 (22.9%)	120 (23.3%)	510 (23.0%)
Missing	23	1	24
VPAI total score, mean (SD)	31.50 (6.67)	32.43 (6.60)	31.71 (6.67)

### Pre-disclosure VPAI

The VPAI items endorsed most frequently overall were “the desire to contribute to research on AD” (89.5%), followed by “to know more about my risk of developing AD dementia” (80.8%) (Table 3). For both amyloid groups, “confirming the feeling that I might already be developing symptoms of AD dementia” (25.3%) was the least frequently endorsed item.

**Table 3:** Pre-disclosure response of participants endorsing items included in the views and perceptions of amyloid imaging questionnaire at the two highest levels (“very” or “extremely” important)

	**Not Elevated (N=1724)**	**Elevated (N=517)**	**Total (N=2241)**	**adj p value**
Seek info preventative measures	1219 (70.7%)	371 (71.8%)	1590 (71.0%)	>0.999
To put mind at ease	1145 (66.5%)	366 (70.8%)	1511 (67.5%)	0.413
Know risk of developing dementia	1379 (80.0%)	431 (83.4%)	1810 (80.8%)	0.49
Able to participate in trials	1314 (76.2%)	401 (77.6%)	1715 (76.5%)	>0.999
Contribute to Alzheimer’s research	1540 (89.3%)	466 (90.1%)	2006 (89.5%)	>0.999
Arrange my personal affairs	539 (31.3%)	178 (34.4%)	717 (32.0%)	0.716
Feel I might be developing dementia	400 (23.2%)	166 (32.1%)	566 (25.3%)	<0.001
Prepare family for possible illness	623 (36.1%)	229 (44.3%)	852 (38.0%)	0.008
Curiosity	773 (44.8%)	206 (39.9%)	979 (43.7%)	0.341

Note: Chi-squared tests with holm adjustments were used to calculate p-values

Participants in the elevated amyloid group had higher endorsements than participants in the not elevated amyloid group for eight out of the nine VPAI items (Table 3). The proportion of participants who responded at the two highest levels of endorsement were statistically greater in the elevated amyloid group compared to the not elevated amyloid group in two items, “to confirm the feeling that I might already be developing symptoms of AD dementia” (p<0.001) and “to prepare my family for my possible illness in the future” (p=0.008).

**Figure 1:**
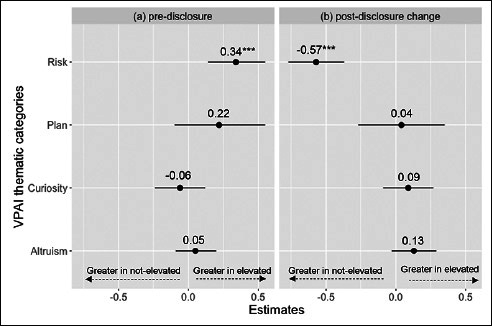
Elevated vs not elevated amyloid group estimates across all models for (a) pre-disclosure and (b) change (post – pre disclosure) The VPAI items included in each thematic category are presented in Table 1.

In our secondary analyses of the pre-disclosure thematic category scores, perceived risk was the only category that showed a significant difference between the elevated and not elevated amyloid groups (Figure 1a). Participants with elevated amyloid had a higher perceived risk score than did the not elevated amyloid group (est: 0.34; CI:0.14, 0.55; p=0.001). Female sex was associated with higher scores in all categories. Having a family history of AD was associated with higher perceived risk, altruism/contribute, and plan/prepare scores. Older age was associated with higher perceived risk and plan/prepare category scores (data available upon request).

**Table 4:** Categorized change in the binary VPAI item score (post disclosure - pre disclosure) by amyloid group. Changes in score are presented as Reduced (−1), No Change (0) or Increased (1)

	**Not Elevated (N=1724)**	**Elevated (N=517)**	**Total (N=2241)**
Seek info preventative measures			
Missing	0	1	1
Reduced	296 (17.2%)	68 (13.2%)	364 (16.2%)
No change	1207 (70.0%)	375 (72.7%)	1582 (70.6%)
Increased	221 (12.8%)	73 (14.1%)	294 (13.1%)
To put mind at ease			
Missing	1	2	3
Reduced	169 (9.8%)	100 (19.4%)	269 (12.0%)
No change	1288 (74.8%)	353 (68.5%)	1641 (73.3%)
Increased	266 (15.4%)	62 (12.0%)	328 (14.7%)
Know risk of developing dementia			
Reduced	158 (9.2%)	51 (9.9%)	209 (9.3%)
No change	1398 (81.1%)	426 (82.4%)	1824 (81.4%)
Increased	168 (9.7%)	40 (7.7%)	208 (9.3%)
Able to participate in trials			
Reduced	193 (11.2%)	52 (10.1%)	245 (10.9%)
No change	1304 (75.6%)	390 (75.4%)	1694 (75.6%)
Increased	227 (13.2%)	75 (14.5%)	302 (13.5%)
Contribute to Alzheimer’s research			
Reduced	113 (6.6%)	30 (5.8%)	143 (6.4%)
No change	1492 (86.5%)	454 (87.8%)	1946 (86.8%)
Increased	119 (6.9%)	33 (6.4%)	152 (6.8%)
Arrange my personal affairs			
Reduced	163 (9.5%)	59 (11.4%)	222 (9.9%)
No change	1295 (75.1%)	376 (72.7%)	1671 (74.6%)
Increased	266 (15.4%)	82 (15.9%)	348 (15.5%)
Feel I might be developing dementia			
Reduced	133 (7.7%)	56 (10.8%)	189 (8.4%)
No change	1296 (75.2%)	377 (72.9%)	1673 (74.7%)
Increased	295 (17.1%)	84 (16.2%)	379 (16.9%)
Prepare family for possible illness			
Reduced	172 (10.0%)	78 (15.1%)	250 (11.2%)
No change	1226 (71.1%)	364 (70.4%)	1590 (71.0%)
Increased	326 (18.9%)	75 (14.5%)	401 (17.9%)
Curiosity			
Missing	2	1	3
Reduced	240 (13.9%)	57 (11.0%)	297 (13.3%)
No change	1214 (70.5%)	359 (69.6%)	1573 (70.3%)
Increased	268 (15.6%)	100 (19.4%)	368 (16.4%)

### Post-disclosure VPAI change

The interaction between amyloid group and time (pre- and post-disclosure VPAI item response) in the GEE models was significant for four items. The elevated amyloid group had higher odds of positive change for the item “curiosity” (OR: 1.32; p=0.014). The not elevated amyloid group had higher odds of positive change in categorical VPAI item level scores for the items “to confirm the feeling that I might already be developing symptoms of AD dementia” (OR: 0.79; p=0.049), “to prepare my family for my possible illness in the future” (OR: 0.67; p=<0.001), and “to put mind at ease” (OR: 0.54; p<0.001). The pre- and post-disclosure probabilities of participants scoring a categorical VPAI item level of 1 (“very” or “extremely” important) for the four items can be found in Supplemental Table 1.

In our analyses of thematic category change scores, participants in the not elevated amyloid group had a greater change in perceived risk score compared to the elevated amyloid group (est: −0.57; CI: −0.77, −0.37; p<0.001). We did not observe statistically significant differences between amyloid groups in change scores for the other three thematic categories (Figure 1b). Older age was associated with a lower change in scores for the altruism/contribute (est: −0.02; CI: −0.03, −0.01; p=0.004) and plan/prepare categories (est: −0.04; CI: −0.06, −0.01; p=0.002) (data available upon request).

## Discussion

Preclinical AD has been demonstrated as a feasible disease stage in which to test potential disease-slowing therapies (16). It will be important to continue work to improve the design and conduct of these trials, including participant recruitment and retention. While many papers on willingness to participate in trials report results from hypothetical scenario studies, here we report results from actual participants. In particular, we examined data from one preclinical AD trial to better understand the motivations of participants to undergo biomarker testing and enroll in these trials.

Recruitment of participants is a major challenge in AD trials. Inadequate recruitment increases the financial cost of trials but may also result in late detection of safety signals, prolonged or terminated trials, and delayed access to treatment (17–19). Incorporating participant-centric strategies into the recruitment framework, therefore, may be a key step toward efficient accrual. Consistent with previous findings (7, 8), our study found that in both amyloid groups, participants most often endorsed the desire to contribute to research as a motivation to participate. Emphasizing altruism and contributing to research progress, therefore, appear to be essential to emphasize in future trial recruitment strategies. For example, trialists could modify recruitment material to focus on how enrollment can contribute to advancing scientific research, emphasizing the specific goals of each study whenever possible.

Retaining participants until study completion is equally important to ensure trial integrity (18, 20). Greater than anticipated dropout can impact the precision of the estimated treatment effects. It could also lead to biased results if participants with certain characteristics are at disproportionately high risk to drop out of the trial. Similar to recruitment, trialists may need to consider implementing retention strategies that align with participant reported altruistic motivations to undergo biomarker testing and disclosure. Reminding participants of the value of their continued contribution to advancing science may be an effective retention intervention that appeals to participants’ altruistic motivations. Future research should assess whether the responses on the VPAI are also associated with the outcome of trial completion.

Most important to preclinical AD trial recruitment is the enrollment of those eligible for randomization, that is, participants with elevated amyloid. While previous studies have reported that disclosure in the setting of preclinical AD trials is safe and is not a barrier to enrollment (21–24), a large number of individuals must undergo screening to identify an adequate number of eligible participants. Screening using CSF is invasive and PET scans are costly (25). While recent advances in blood-based biomarkers hold promise as a cost-effective tool to identify amyloid eligible participants (26), a high proportion of participants will still be excluded. Among those who underwent biomarker testing, approximately 70% of participants in the A4 study (17) and 77% of participants in the EARLY study were excluded based on their biomarker results. Given that amyloid testing remains a unique requirement of participating in preclinical AD trials, prioritizing recruitment strategies that target motivating factors in the VPAI endorsed by participants with elevated amyloid may yield a more efficient approach to recruit individuals who will be eligible. We observed generally higher endorsement for the VPAI items in participants with elevated compared to those with not elevated amyloid before they underwent biomarker testing and learned their result. In the EARLY trial, the proportions of participants endorsing confirm feeling that I might be developing dementia (p<0.001) and preparing family members (p=0.008) were observed to be higher among participants with elevated compared to not elevated amyloid. Similarly, in our analyses of thematic categories, the elevated amyloid group had a significantly higher perceived risk score compared to the not elevated amyloid group. Previous studies have found that participants and their study partners who report greater subjective cognitive complaints were at higher risk of having elevated levels of brain amyloid (27) and disease progression (28, 29). Therefore, targeting individuals with subjective cognitive complaints and emphasizing the opportunity to learn more about their concerns and plan for the future may be an important component of recruitment strategies designed to target those most likely to be eligible for preclinical AD trials.

In our pre-disclosure analyses of thematic category scores, some participant characteristics were associated with higher endorsement of VPAI items. For example, we found that having a family history of AD was associated with higher scores in perceived risk, altruism/contribute and plan/prepare. We also found that female sex was associated with higher VPAI in all four thematic categories and older age was associated with higher perceived risk and plan/prepare category scores. These results parallel studies demonstrating that females and older participants may be more willing to enroll in AD trials (7). While implementing an upper age limit in preclinical AD trials can reduce the risk of enrolling participants with other comorbid pathologies, trialists should also consider that such age limits might prevent participation by potentially eligible participants who are more likely to have elevated amyloid and better represent the age group suffering from dementia (30).

In exploratory analyses, we observed some differences between amyloid groups in VPAI change scores following disclosure. We found that the interaction between amyloid group and time (pre- vs. post-disclosure VPAI item level score) was significant for putting mind at ease; participants in the not elevated amyloid group showed an increase in endorsement. This result aligns with observations in the Study of Knowledge and Reactions to Amyloid Testing (SOKRATES), a qualitative study of individuals learning their biomarker status in the A4 study. In SOKRATES, 63% of participants learning a not elevated result felt relieved about their futures, while no participants with elevated amyloid expressed a sense of relief (31). Additionally, we observed a reduction in endorsement of this item post-disclosure among the participants with elevated amyloid. This may further suggest that for some participants in the elevated amyloid group, the importance of putting their mind at ease may diminish after learning their elevated amyloid status. For both amyloid groups, there was an increase in endorsement for the item feel I might be developing dementia, with higher odds of positive change observed in the not elevated amyloid group. While amyloid disclosure may have validated subjective memory concerns for the elevated amyloid group, participants in the not elevated amyloid group may have viewed the item as more important post-disclosure as it reassured them that they have not developed AD and provided them with the opportunity to re-interpret their subjective cognitive complaints as a normal part of aging (31). Surprisingly, more participants in the not elevated amyloid group showed an increase in endorsement for preparing family members compared to the elevated amyloid group. When learning their amyloid results, participants in the not elevated amyloid group may have desired to share the negative results with their family members, while those in the elevated amyloid group may have been more inclined to focus on individual level factors (i.e., what comes next). For example, in one study of biomarker disclosure, the relatively few participants who expressed uncertainty about sharing their biomarker results cited being selective about with whom to share results, wanting more information before sharing, and feeling a sense of ambiguity about prognostic implications (32). Some participants may also feel reluctant to share their results with family members due to stigma associated with AD (31). Finally, we observed a significant interaction between amyloid group and time for curiosity item. Participants in the elevated amyloid group may have expressed greater change in importance for this item post-disclosure as they may have become more aware or concerned about their memory problems after learning their result (31).

In our examination of thematic category change scores, we observed that for the perceived risk category, participants in the not elevated amyloid groups demonstrated significantly greater change in scores compared to the elevated amyloid group (est: −0.57; CI: −0.77, −0.37; p<0.001), similar to the findings from the thematic category results in the A4 trial (11). Unlike the analyses of VPAI from the A4 Study, however, we did not observe significant differences between amyloid groups in the pre/post change scores in the plan/prepare and altruism/contribute categories (8). It is possible that these inconsistencies could have been driven by differences in study design between the A4 and EARLY trials. While the A4 trial completed biomarker testing by PET scan alone, the EARLY trial screened participants by PET, CSF, or both. Previous studies have found that more participants are willing to engage in studies involving PET scans compared to lumbar punctures (33), and some individuals fear the invasiveness and potential adverse events associated with lumbar punctures (34). Thus, use of CSF may have caused differential sample bias between the trials. Additionally, a recent study showed that participants have higher confidence in their AD diagnosis when the clinical workup includes brain scans as compared to other assessments (35) and previous analyses of the EARLY trial disclosure data suggested potential differences in reactions to biomarker information based on the modality of the test (36). Finally, the EARLY trial also had a greater geographic representation compared to the A4 trial, including recruitment from more countries, perhaps introducing important cultural variations (36, 37).

There were limitations to this study. There is sample bias related to willingness to undergo biomarker testing and to enroll in a drug trial. Additionally, cognitive testing and clinical assessments during the screening process further narrowed the selection of participants from the target population (1). Beyond these identified biases, there are also unknown factors from selection bias that may have influenced our findings. We did not report differences in subjective cognitive concerns between amyloid groups or adjust our analyses for subjective cognitive concerns given that approximately 80% of the participants in our sample were missing the Cognitive Function Instrument data. While we based the thematic VPAI categories on a previously published paper (8), we cannot rule out that different assignment of the VPAI items could have produced different results. Furthermore, we cannot be certain that they align with every participant’s use of the scale. For example, some participants may have endorsed joining the preclinical AD trial out of a desire to gain access to a perceived promising therapy rather than to contribute to science (item #4). Interpreting change scores also presents challenges as there is no gold standard approach to utilize the VPAI scale data. As a result, we considered the assessments of change as exploratory, aimed at generating hypotheses. As our analyses of change in VPAI were exploratory, we did not adjust the models for multiple comparisons. Additional studies are needed to elucidate the factors that contributed to the variations in some of the item-level change scores between the two amyloid groups. The EARLY trial also enrolled a relatively low proportion of non-White and Hispanic participants, which prevented specific subgroup comparisons. Future studies should examine potential differences among subpopulations as well as potential regional differences in motivation to undergo amyloid imaging in preclinical AD trials (38). Finally, we are unable to assess whether and how participants could have changed their approach to completing the follow-up VPAI scale. The scale asks about reasons to undergo the biomarker test. After biomarker testing and learning their result participants may have reinterpreted the VPAI questions based on their new context (i.e., the impact of the test result) or they may have reassessed their motivations to undergo testing at baseline.

## Conclusion

Consistent with previous findings, we observed that altruism was a key motivating factor for participants enrolled in the EARLY trial. Before biomarker testing and disclosure, participants in the elevated amyloid group showed higher endorsement of the VPAI items than the participants in the not elevated amyloid group. Future trials may want to adjust recruitment strategies to emphasize motivations endorsed by participants in the elevated amyloid group.

## Supplemental Material

Supplementary material, approximately 16 KB.

## Notes

*Ethical standards*: All participants in the EARLY trial signed an IRB-approved consent form. This included consent to secondary analyses such as those included in this manuscript. This study did not meet the criteria for human subjects research, as no identifiable information was used.
