# Supplementary material for: Views and Perceptions of Amyloid Imaging in a Preclinical Alzheimer’s Disease Trial
Source: J Prev Alzheimers Dis. 2024 Sep 3;11(6):1563–71. doi: 10.14283/jpad.2024.157 (PMC11573807; doi:10.14283/jpad.2024.157)
Supplement: Supplementary file 1 — Supplementary material, approximately 16 KB. [file 42414_2024_157_MOESM1_ESM.docx]

SUPPLEMENTAL MATERIAL

Supplemental Table 1. The pre- and post-disclosure probabilities of participants scoring a categorical VPAI item level of 1 (“very” or “extremely” important) for the four items with significant interactions between amyloid group and time in the GEE models.

| VPAI Items | Amyloid group | Pre-disclosure | Post-disclosure |
| --- | --- | --- | --- |
| To put mind at ease | Not elevated | 0.66 | 0.72 |
|  | Elevated | 0.69 | 0.62 |
| Feel I might be developing dementia | Not elevated | 0.23 | 0.33 |
|  | Elevated | 0.30 | 0.35 |
| Prepare family for possible illness | Not elevated | 0.36 | 0.45 |
|  | Elevated | 0.42 | 0.42 |
| Curiosity | Not elevated | 0.44 | 0.46 |
|  | Elevated | 0.39 | 0.47 |
